# Treatment of Influenza with Camphor

**Published:** 1892-01

**Authors:** 


### TREATMENT OF INFLUENZA WITH CAMPHOR.

During the recent epidemic, when I had on an average of 150 cases a week under my care, I had ample opportunity of testing the efficacy of various methods of treatment. My fisst idea was to try to alleviate the most prominent symptoms—namely, backache and general pains, with sod. salicyl; headache, etc., with analgesin, combining either of the above with a sedative or stimulating expectorant if the chest was affected. I also used ergot and digitalis in combination in a few cases, believing the disease to be simptly due to a vaso-motor change, but I afterward came to regard it as a zymotic disease, with probably a special bacillus of its own. Amongst other drugs I tried camphor, and with so much success that I rarelv prescribed anything else afterward, six doses or less usually being sufficient. I administered it as follows :

| | | |
|---|---|---|
| ℞ | Sp. Camph., - | dr. ij. |
| | Tinct. lavand co. - | dr. ij. |
| | Sp. chlorof. - | dr. j. |
| | Mucilag. tragacanth | oz. ij. |
| | Aq. ad. - | oz. vj. |

Oz. j quartis horis sumend.

This cost very little, and, by leaving out the flavoring agents the effect is the same and the cost nominal.—*Long, British Medical Journal,—St. Louis Clinique.*

